# Supplementary material for: How antisolvent miscibility affects perovskite film wrinkling and photovoltaic properties
Source: Nat Commun. 2021 Mar 10;12:1554. doi: 10.1038/s41467-021-21803-2 (PMC7946869; doi:10.1038/s41467-021-21803-2)
Supplement: Supplementary file 1 — Supplementary Information [file 41467_2021_21803_MOESM1_ESM.pdf]

Supplementary Information

How antisolvent miscibility affects perovskite film wrinkling and photovoltaic properties

Seul-Gi Kim[1], Jeong-Hyeon Kim[1], Philipp Ramming[2,3], Yu Zhong [2,3], Konstantin Schötz[3], Seok Joon Kwon[1,4], Sven Huettner[2], Fabian Panzer[3], Nam-Gyu Park[1]*

[1]School of Chemical Engineering, Sungkyunkwan University (SKKU), Suwon 440-746, Republic of Korea.

[2]Department of Chemistry, University of Bayreuth, Universitätsstrasse 30, 95447 Bayreuth, Germany.

[3]Chair for Soft Matter Optoelectronics, University of Bayreuth, Universitätsstrasse 30, 95447 Bayreuth, Germany

[4]Nanophotonics Research Center, Korea Institute of Science and Technology (KIST), Seoul 02792, Korea.

*Corresponding author

E-mail: npark@skku.edu, Tel: +82-31-290-7241

Supplementary Fig. 1-18 : pages s1-s16

Supplementary Table 1-2: pages s17-s18

Supplementary Note 1-4: pages s19-s25

Supplementary References: page s26-s27

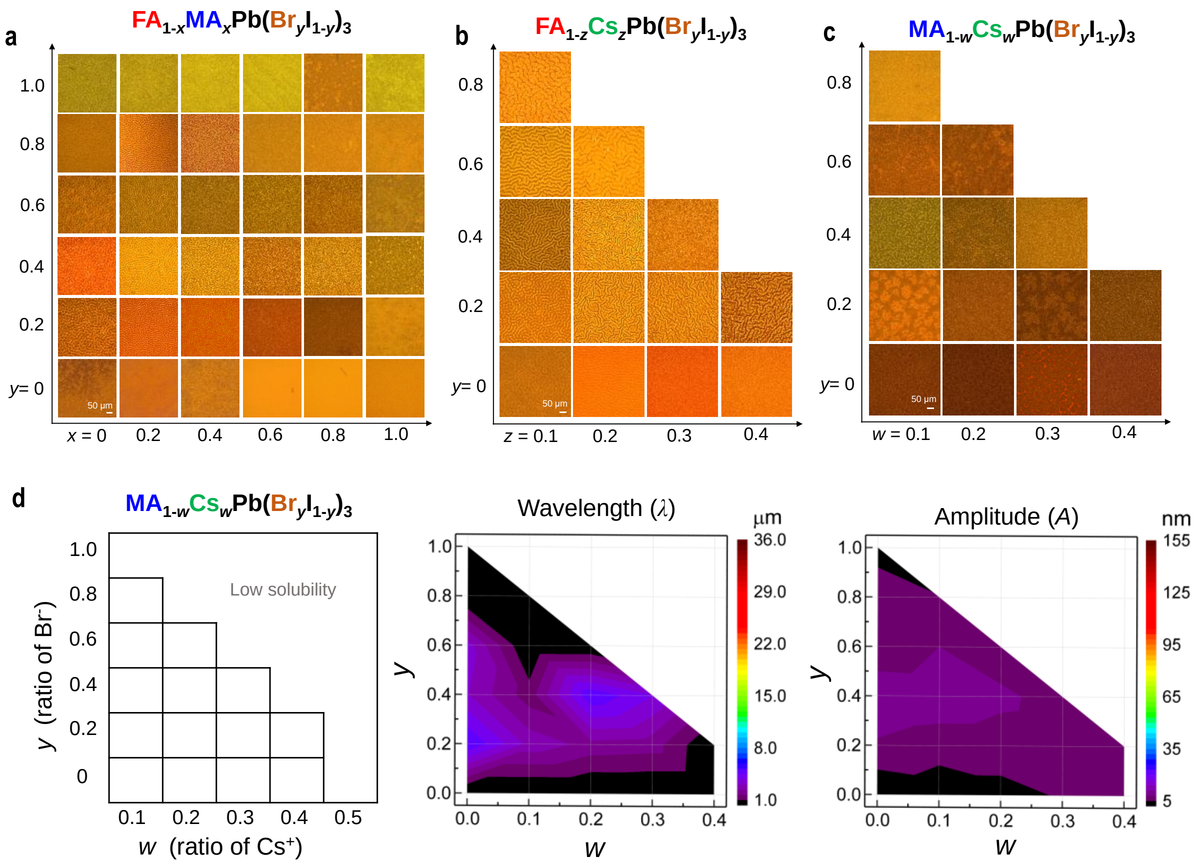

Supplementary Fig. 1. Optical microscope images of the surface morphology of perovskite films with different compositions for (a) $FA_{1-x}MA_xPb(Br_yI_{1-y})_3$, (annealed at 145 °C for 10 min) (b) $FA_{1-z}Cs_zPb(Br_yI_{1-y})_3$ (annealed at 145 °C for 10 min) and (c) $MA_{1-w}Cs_wPb(Br_yI_{1-y})_3$ (annealed at 100 °C for 10 min). (d) Wrinkled phase diagrams of $MA_{1-w}Cs_wPb(Br_yI_{1-y})_3$ perovskite thin films with different compositions, together with color maps showing almost no wrinkled morphologies.

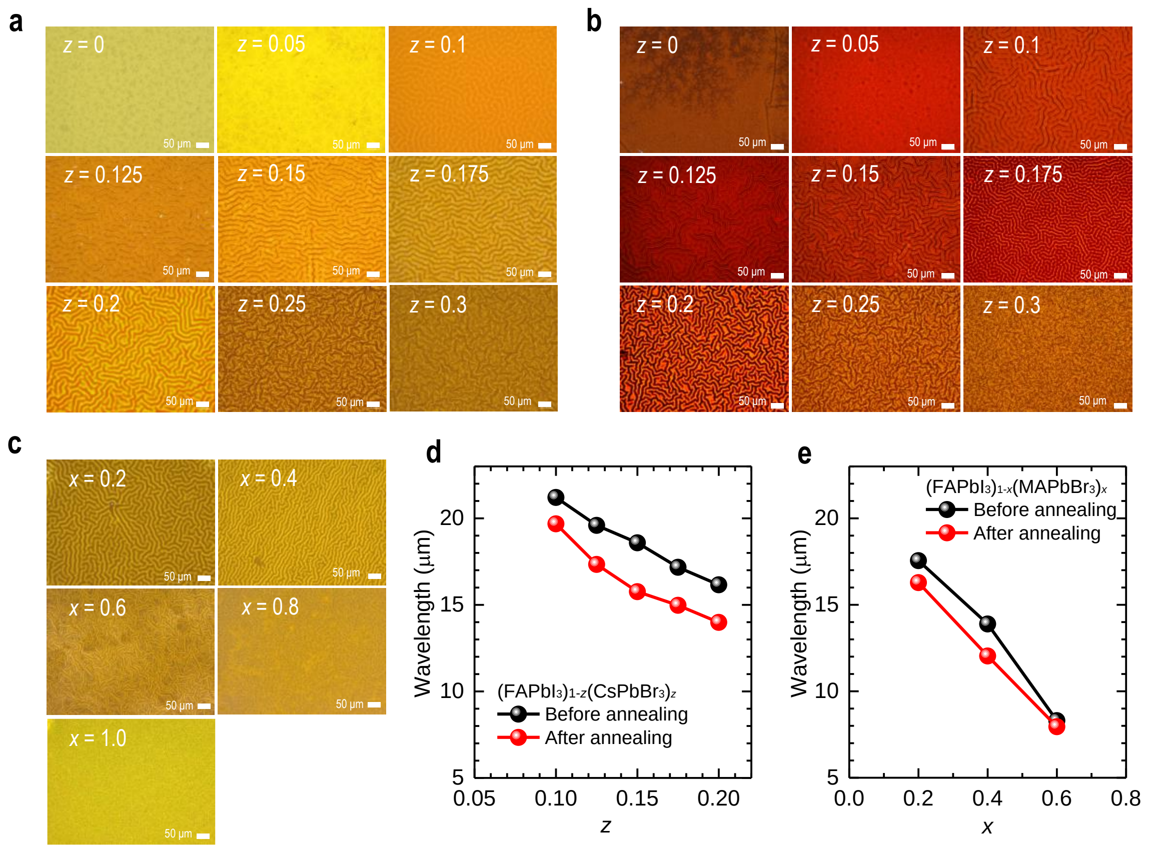

Supplementary Fig. 2. Optical microscope images of the surface morphology of perovskite films with the composition of $(FAPbI_3)_{1-z}(CsPbBr_3)_z$ (a) before and (b) after annealing at 145 °C for 10 min. (c) Optical microscope images of the surface morphology of the non-annealed $(FAPbI_3)_{1-x}(MAPbBr_3)_x$ perovskite film. Effect of the composition on wavelength (λ) of the wrinkled films for (d) $(FAPbI_3)_{1-z}(CsPbBr_3)_z$ and (e) $(FAPbI_3)_{1-x}(MAPbBr_3)_x$ perovskite film before and after annealing. All the images were obtained with temperature conditions of T_{Sub} = 15 °C and T_{DE} = 15 °C.

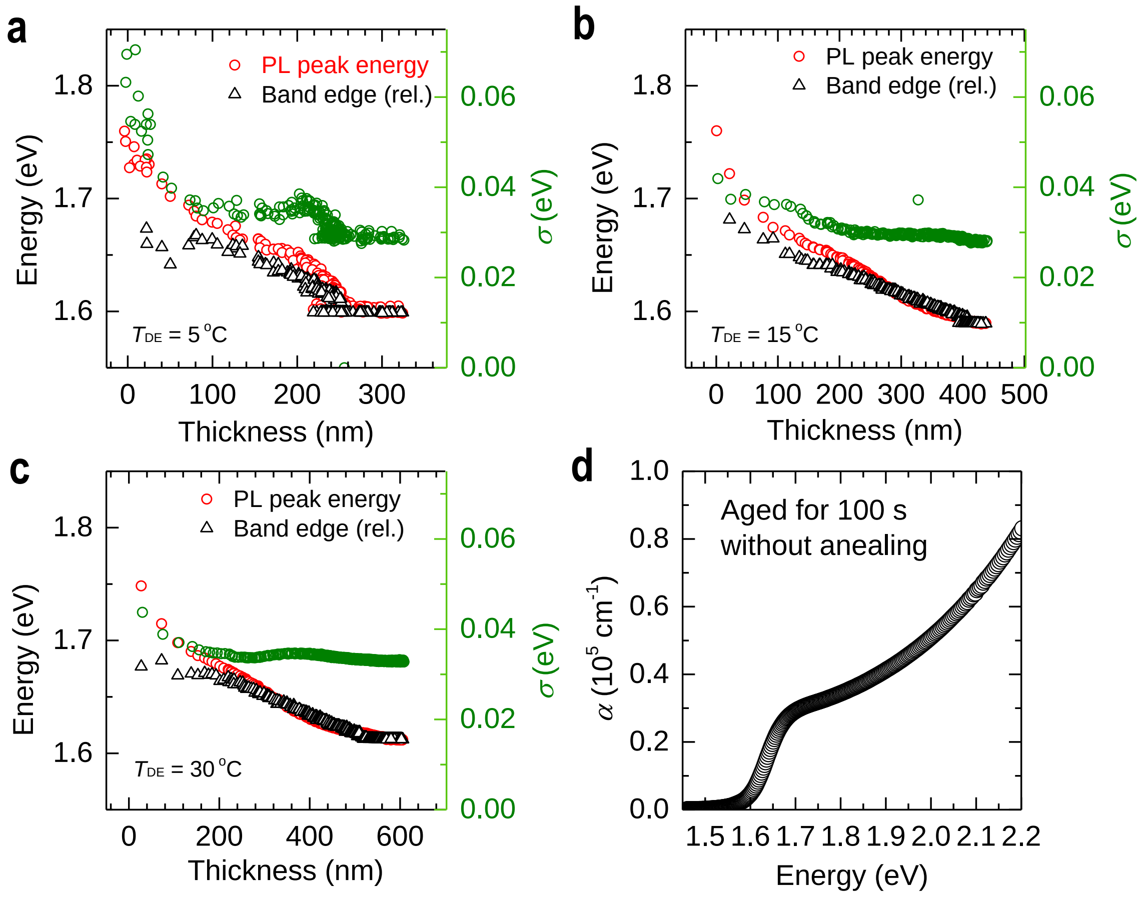

Supplementary Fig. 3. (a-c) PL peak position (red circles), PL peak width σ (green circles), and band edge energy (black triangles) as a function of perovskite layer thickness during spin coating for three different temperatures of diethyl ether. The band edge energy was determined by fitting the band edge of the spectrum at the end of each measurement, to all prior spectra, where the fitted spectrum was allowed to be shifted in energy and scaled in intensity. (d) Absorption coefficient as a function of wavelength for the as-spun film aged for 100 s without annealing. Perovskite composition was $(FAPbI_3)_{0.875}(CsPbBr_3)_{0.125}$.

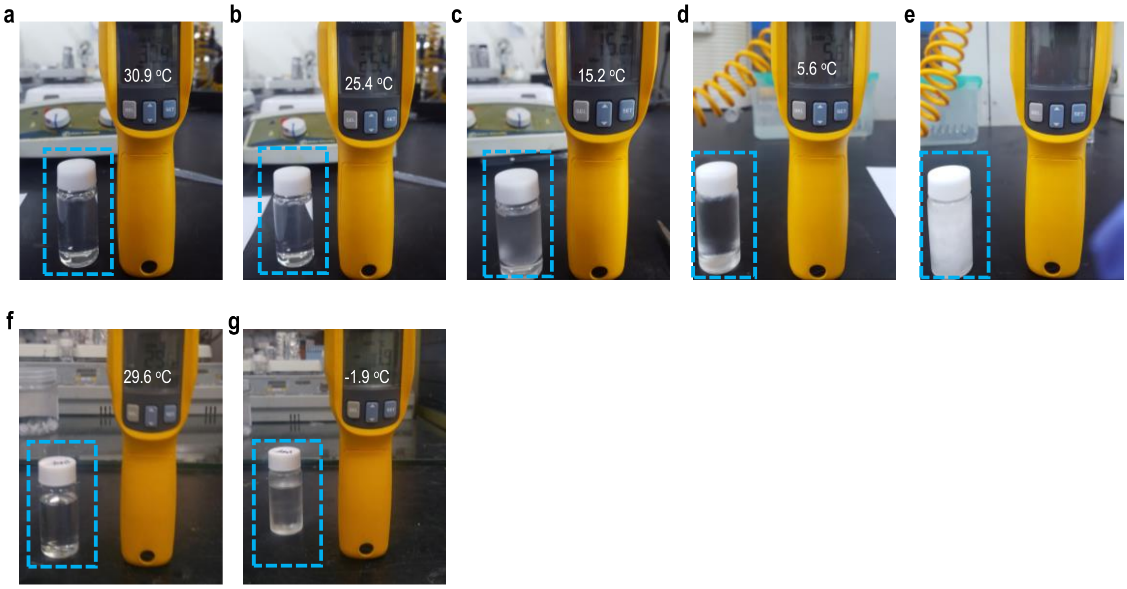

Supplementary Fig. 4. Digital photographs of the mixture of DMSO and diethyl ether (1:8 v/v) at different solution temperatures of (a) 30.9 ºC, (b) 25.4 ºC, (c) 15.2 ºC, and (d) 5.6 ºC. (e) Photograph of the solution prepared at 5.6 ºC showing that the solution was spontaneously frozen. Photographs of the mixture of DMF and diethyl ether (1:8 v/v) at different solution temperatures of (f) 29.6 ºC and (g) -1.9 ºC.

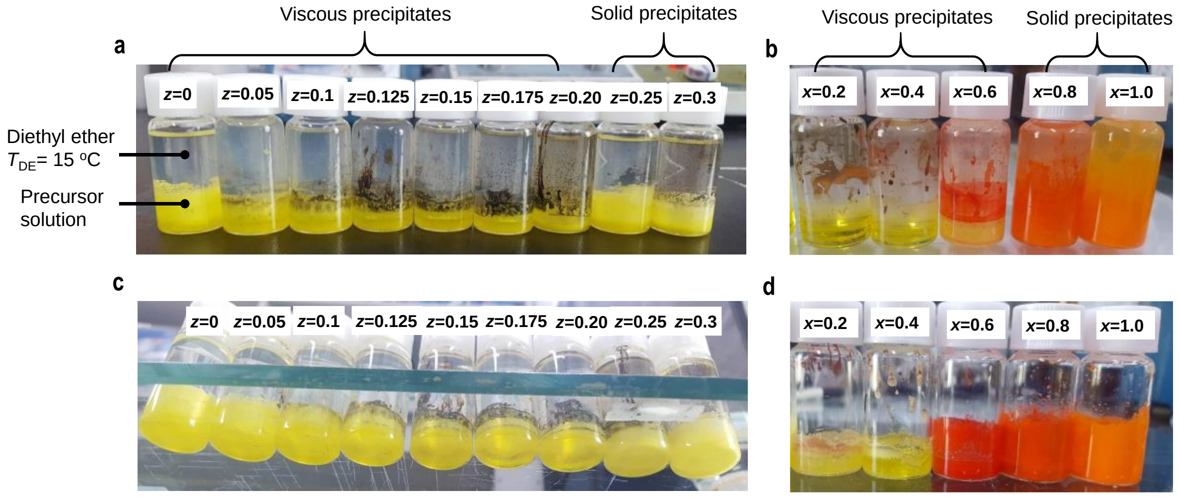

Supplementary Fig. 5. Photographs of the solution formed by pouring diethyl ether (T_{DE} = 15 °C) into a vial containing (a) $(FAPbI_3)_{1-z}(CsPbBr_3)_z$ and (b) $(FAPbI_3)_{1-x}(MAPbBr_3)_x$ precursor solution. The solution was prepared by mixing 1 mL of the precursor solution and 15 mL of diethyl ether, which was followed by extracting the supernatant and then pouring diethyl ether, which was repeated four times to remove DMSO and DMF. The viscous precipitates were formed with range of $0 \leq z \leq 0.2$ for $(FAPbI_3)_{1-z}(CsPbBr_3)_z$ and $0 \leq x \leq 0.6$ for $(FAPbI_3)_{1-x}(MAPbBr_3)_x$ while the precipitates were immediately formed as solid phase for z = 0.25 and 0.3 in $(FAPbI_3)_{1-z}(CsPbBr_3)_z$, x = 0.8 and 1 for $(FAPbI_3)_{1-x}(MAPbBr_3)_x$. In about 5 min, the solutions (a) and (b) were solidified, which was shown in (c) and (d), respectively.

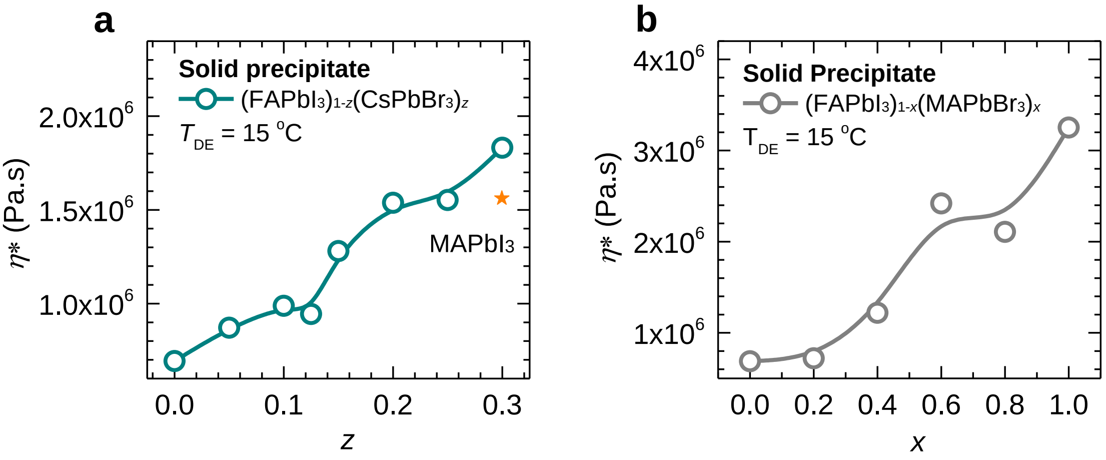

Supplementary Fig. 6. Viscosity of solid precipitates in (a) $(FAPbI_3)_{1-z}(CsPbBr_3)_z$ and (b) $(FAPbI_3)_{1-x}(MAPbBr_3)_x$. The viscosity of solid precipitate of $MAPbI_3$ (star symbol in (a)) was also measured.

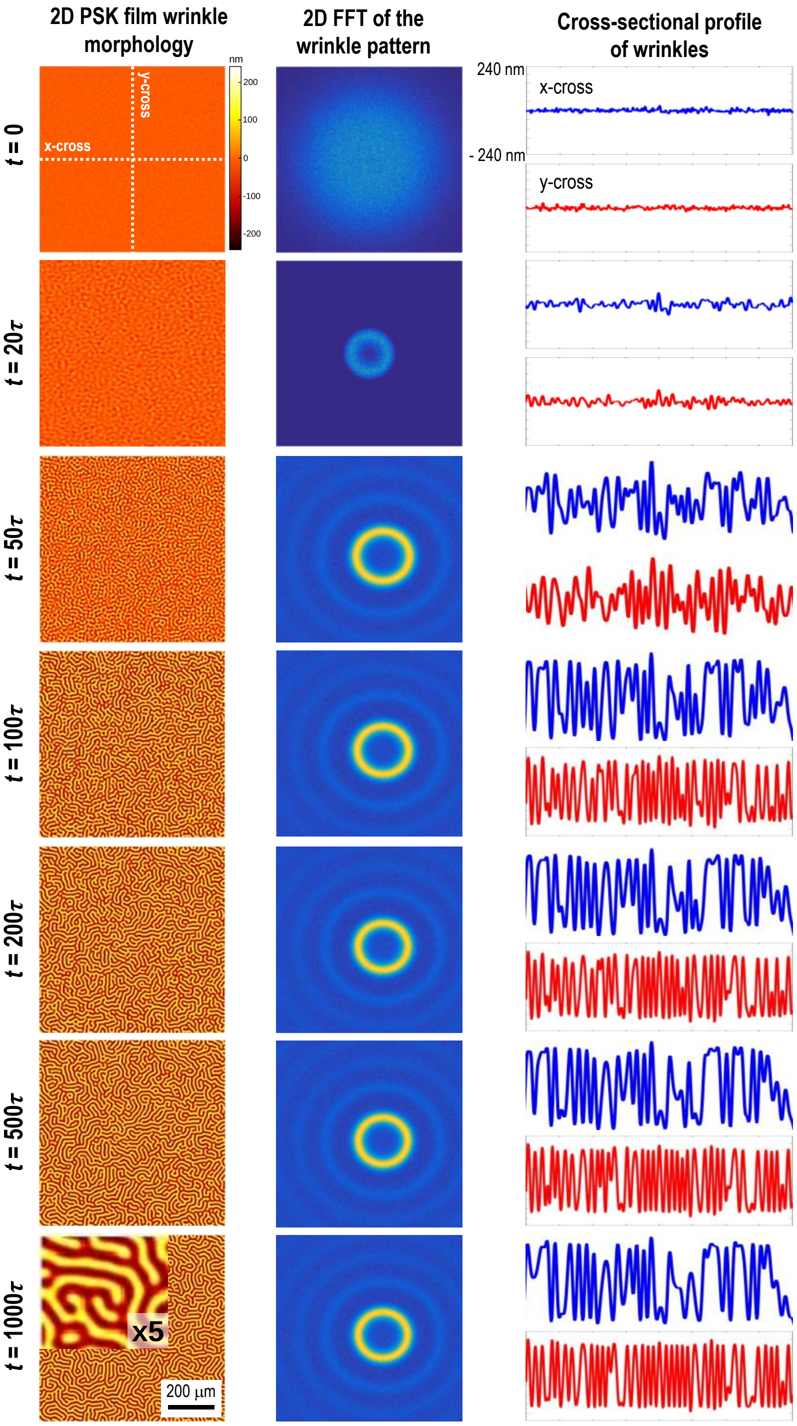

Supplementary Fig. 7. Numerical simulation results of the morphological evolution of perovskite (PSK) wrinkles of elastic-viscoelastic bilayer. τ denotes dimensionless time scale. The left column is for the time-dependent surface wrinkle morphology. The inset for 1000τ shows a 5 times magified image. The middle column is the 2D FFT patterns of the wrinkles. The right column is the cross-sectional profiles of the wrinkles along the horizontal (namely x-cross) and vertical directions (namely y-cross).

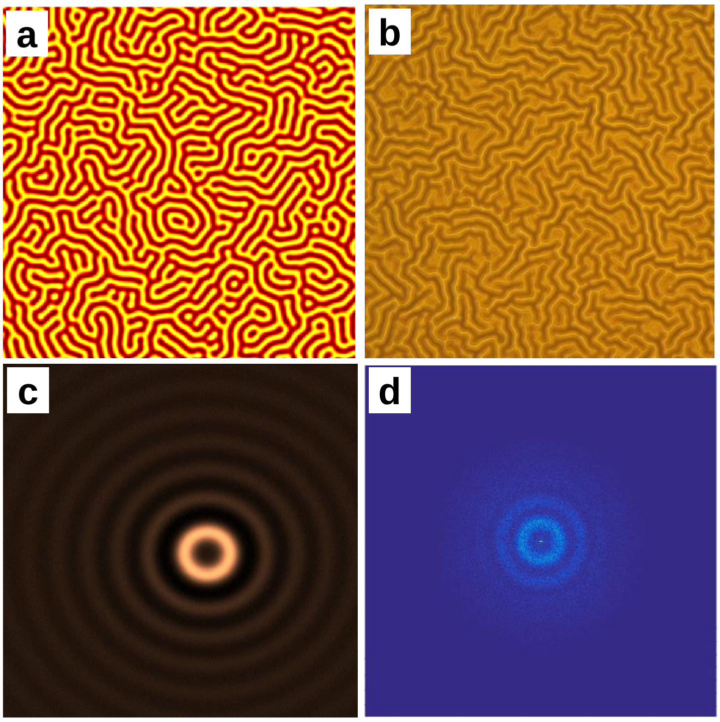

Supplementary Fig. 8. Comparison of the morphologies of the wrinkle pattern obtained from (a) numerical calculation and (b) experimental observation. 2D FFT patterns for (c) numerically calculated wrinkles and (d) experimentally observed wrinkles (c for a and d for b).

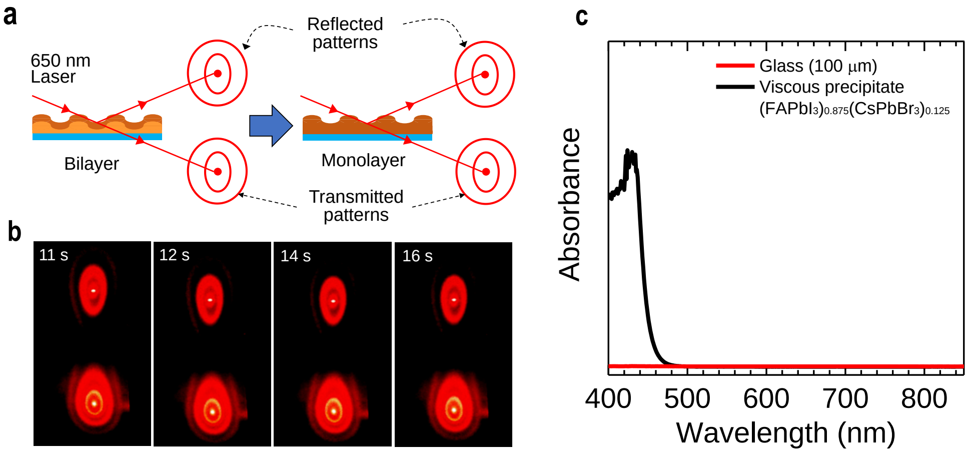

Supplementary Fig. 9. (a) Schematic representation of setup for measuring optical diffraction pattern at top surface of bilayer perovskite film. (b) Photographs of the reflected (up) and the transmitted (down) optical diffraction patterns at top surface as function of spin coating time (11 s, 12 s, 14 s and 16 s) after perovskite precursor was contacted with diethyl ether at 10 s. T_{Sub} = 15 °C and T_{DE} = 5 °C. (c) UV-Vis spectrum of glass substrate and viscous precipitate obtained by pouring diethyl ether to the $(FAPbI_3)_{0.875}(CsPbBr_3)_{0.125}$ perovskite precursor solution.

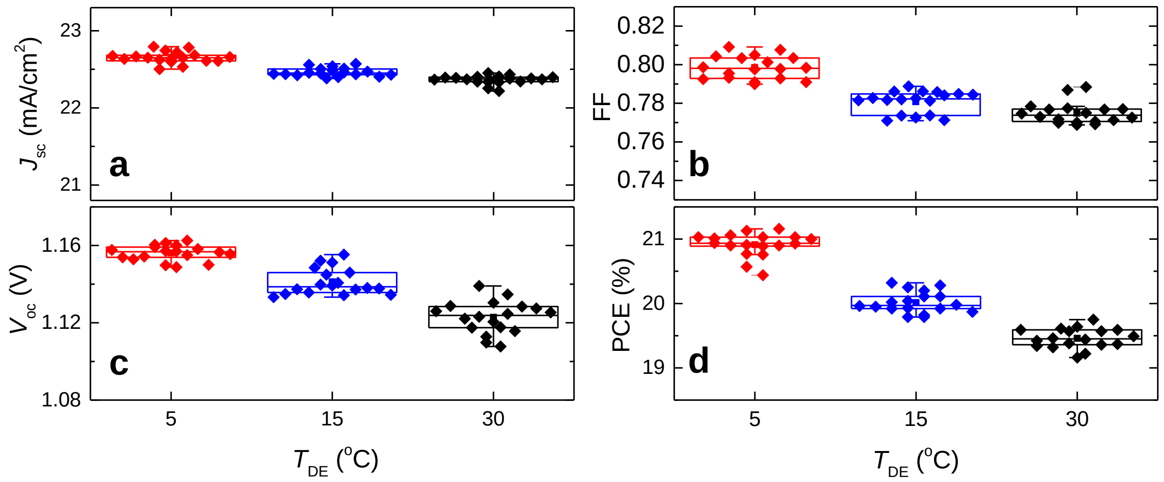

Supplementary Fig. 10. Statistical photovoltaic parameters of (a) J_{sc}, (b) FF, (c) V_{oc} and (d) PCE for the $(FAPbI_3)_{0.875}(CsPbBr_3)_{0.125}$ PSCs as function of T_{DE} (5 °C, 15 °C and 30 °C), measured at a scan rate of 130 mV/s under A.M. 1.5G one sun illumination (100 mW/cm^2). Aperture mask area was 0.125 cm^2.

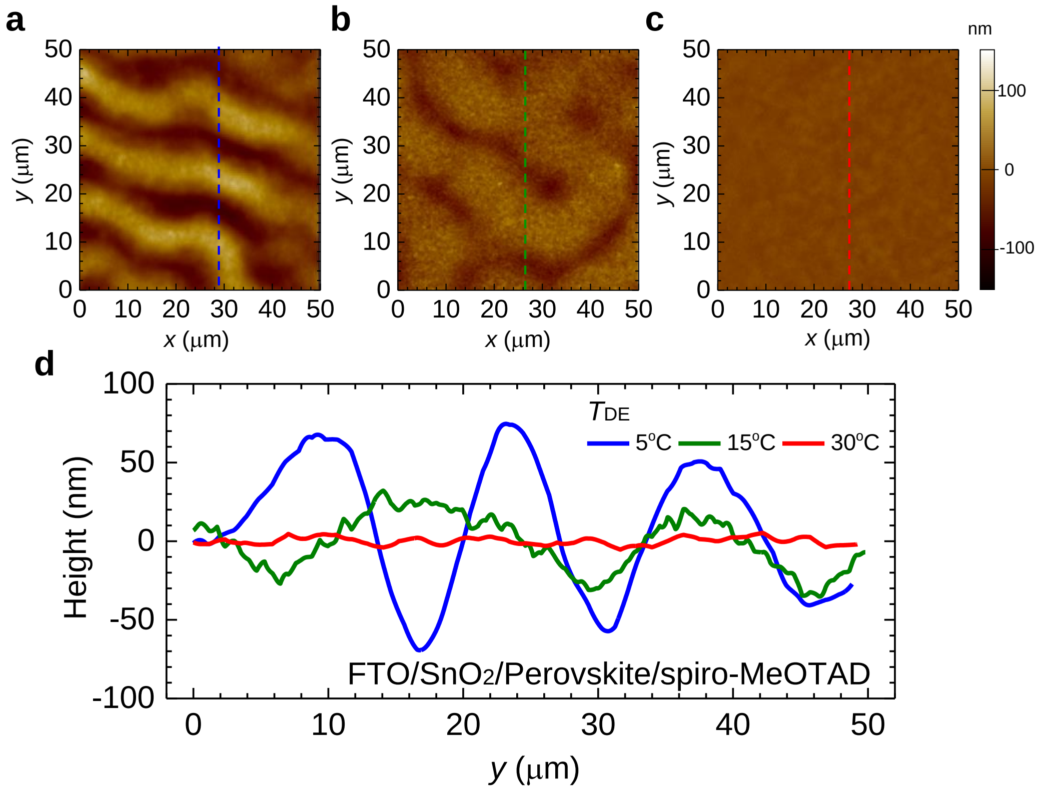

Supplementary Fig. 11. AFM image showing the spiro-MeOTAD surface in the glass/FTO/SnO_2/$(FAPbI_3)_{0.875}(CsPbBr_3)_{0.125}$/spiro-MeOTAD device, where the perovskite layer was formed from T_{DE} = (a) 5 °C, (b) 15 °C and (c) 30 °C. (d) Height profile along the *y*-direction.

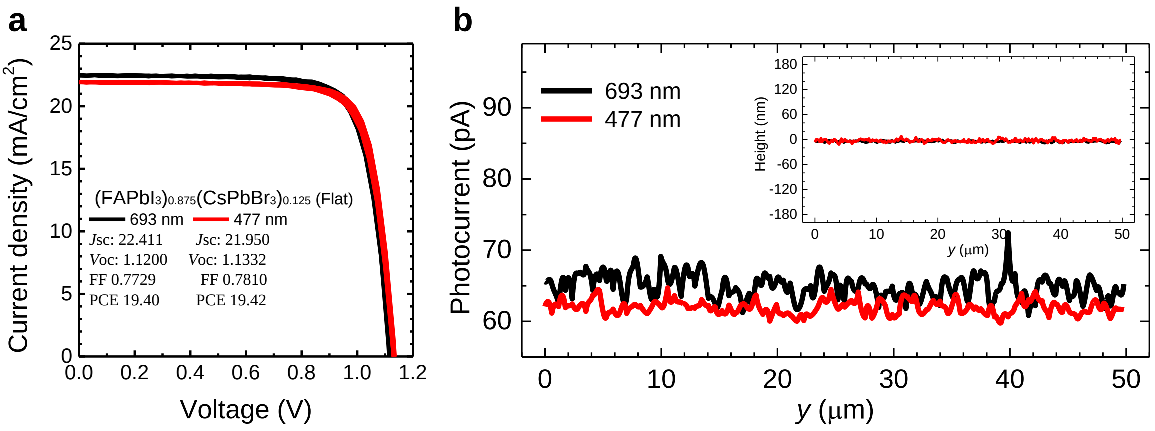

Supplementary Fig. 12. (a) *J-V* curves of PSCs (FTO/SnO_2/Perovskite/Spiro-MeOTAD/Au) employing the flat $(FAPbI_3)_{0.875}(CsPbBr_3)_{0.125}$ films, formed at T_{DE} = 30 °C, with different thickness of 693 nm and 477 nm. (b) Photocurrent profiles of the flat $(FAPbI_3)_{0.875}(CsPbBr_3)_{0.125}$ films (FTO/SnO_2/Perovskite) depending on film thickness. Inset is height profile of $(FAPbI_3)_{0.875}(CsPbBr_3)_{0.125}$ films.

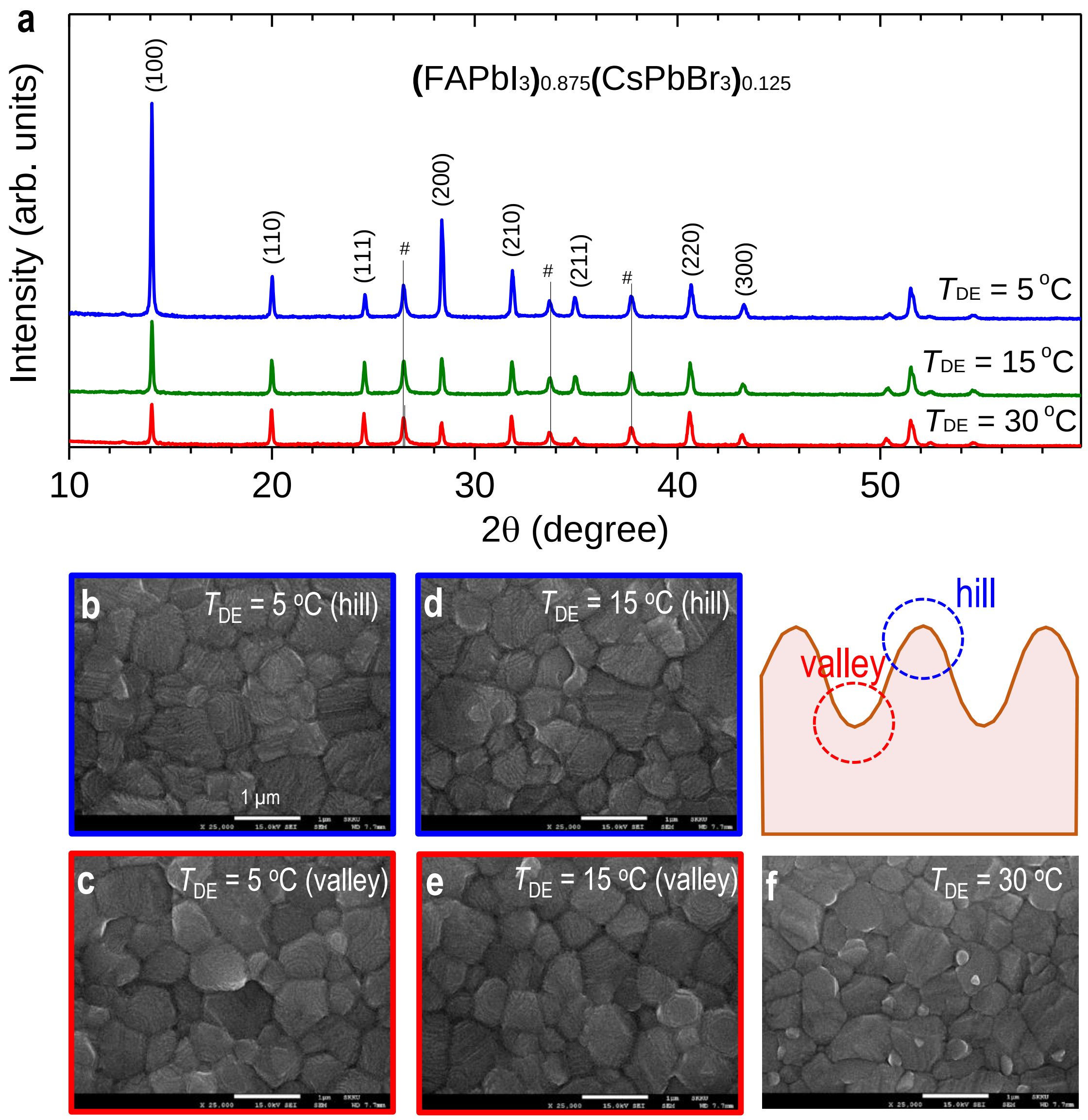

Supplementary Fig. 13. (a) XRD pattern of the $(FAPbI_3)_{0.875}(CsPbBr_3)_{0.125}$ perovskite films formed from T_{DE}= 5 °C, 15 °C and 30 °C. Peaks with # are corresponding to FTO. SEM images of perovskite surface measured at hill and valley region (see cartoon) for T_{DE} = (b, c) 5 °C and (d, e) 15 °C. (f) SEM image of the flat surface formed from T_{DE} = 30 °C.

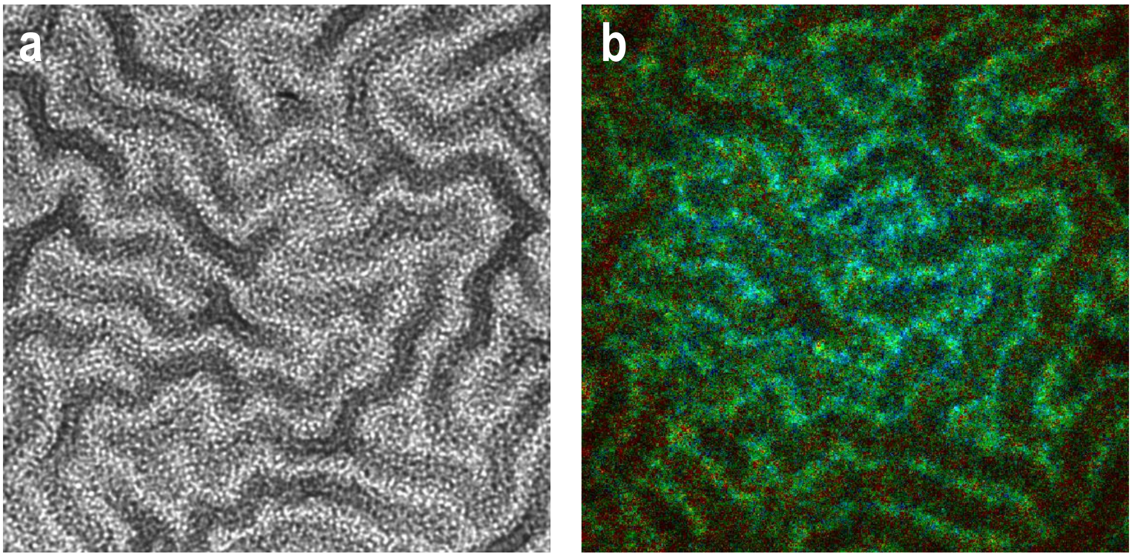

Supplementary Fig. 14. (a) An optical microscope image and (b) the corresponding FLIM image of the $(FAPbI_3)_{0.875}(CsPbBr_3)_{0.125}$ film, taken over the same region (dimension = 100 μm ×100 μm) to identify hills and valleys of the wrinkled morphology.

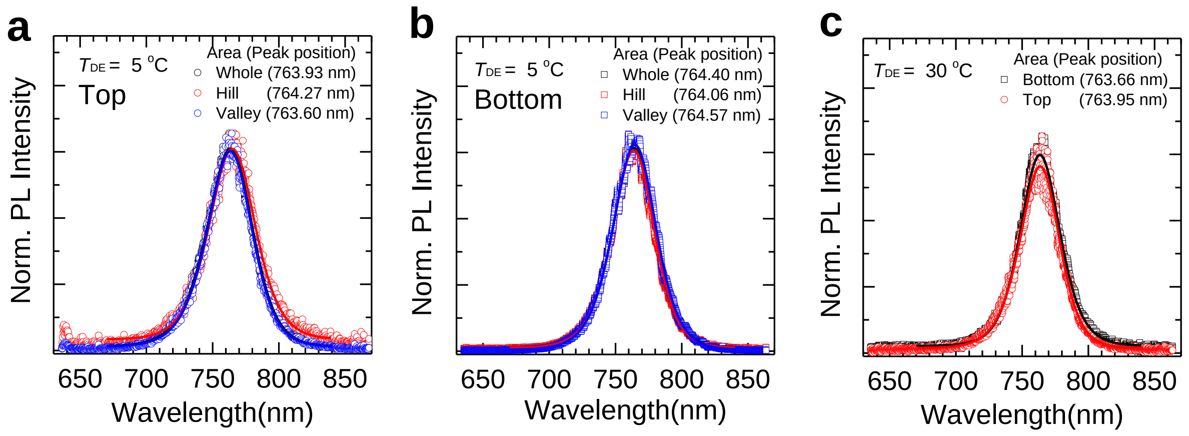

Supplementary Fig. 15. Steady-state PL spectra of (a) the top surface and (b) the bottom surface of the annealed $(FAPbI_3)_{0.875}(CsPbBr_3)_{0.125}$ perovskite film with temperature conditions of T_{Sub} = 15 °C and T_{DE} = 5 °C. (c) PL spectra of top and bottom surface of the annealed $(FAPbI_3)_{0.875}(CsPbBr_3)_{0.125}$ perovskite film with temperature conditions of T_{Sub} = 15 °C and T_{DE} = 30 °C. The surface area for PL measurement was 50×50 μm^2. Since the films formed at T_{Sub} = 15 °C and T_{DE} = 5 °C showed the wrinkled morphology, PL for hill and valley was measured together with the whole film surface area.

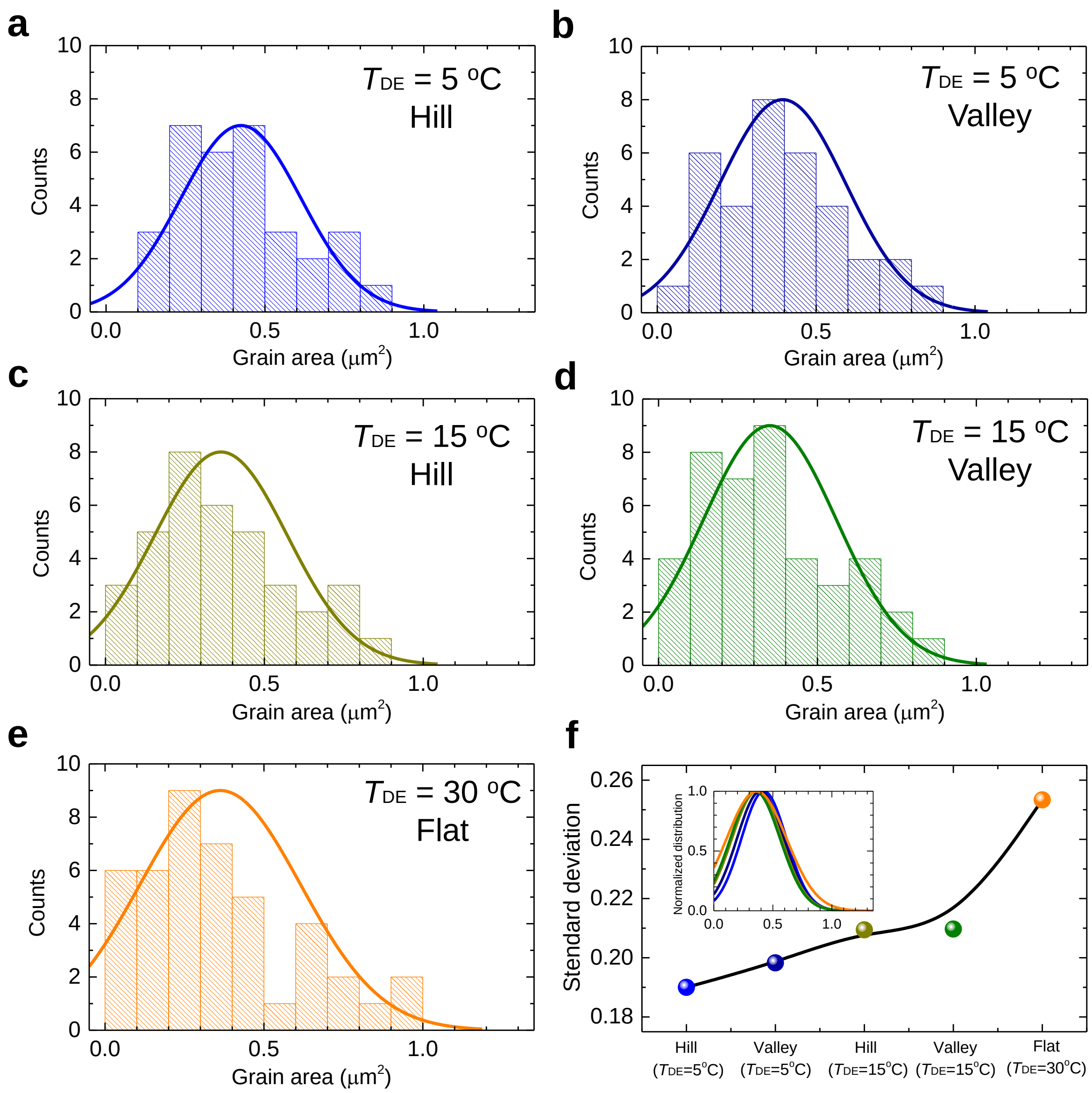

Supplementary Fig. 16. Distributions of grain area (μm^2) extracted from the images in Supplementary Fig. 13, of (a) hill and (b) valley for T_{DE} = 5 °C, (c) hill and (d) valley for T_{DE} = 15 °C and (e) flat for T_{DE} = 30 °C. T_{Sub} was 30 °C. (f) Standard deviation and normalized distribution (inset) of grain size depending on T_{DE}s.

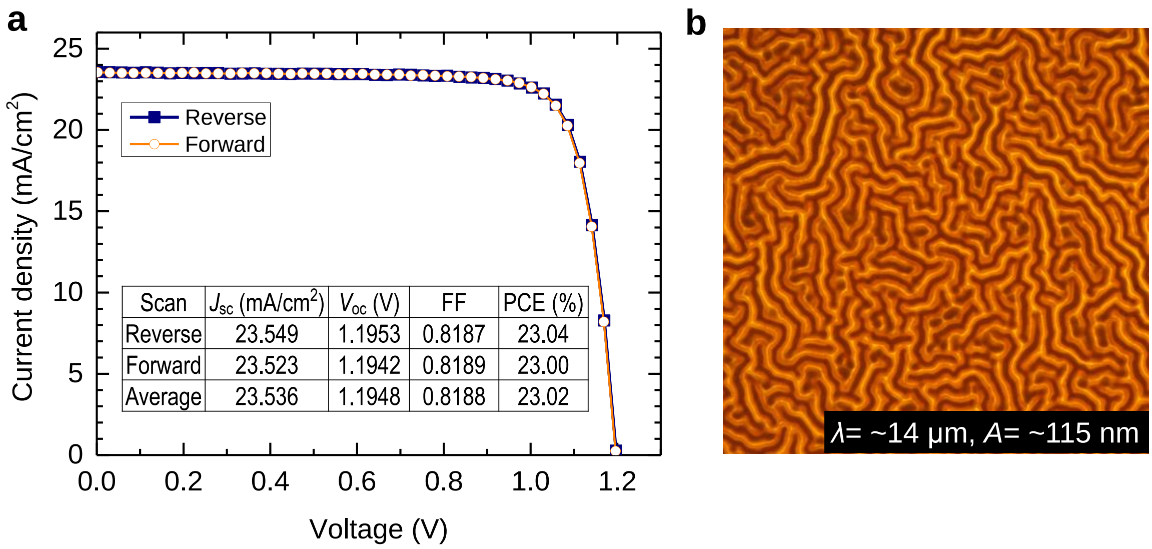

Supplementary Fig. 17. (a) *J*-*V* curve of PSC employing the K-doped $FA_{0.92}Cs_{0.08}PbBr_{0.15}I_{2.85}$ perovskite (T_{Sub} = 15 ºC and T_{DE} = 5 ºC). The ratio of [Pb^{2+}] to [K^{+}] = 0.006. Data were collected under A.M. 1.5G one sun illumination at scan rate of 260 mV/s. Aperture area was 0.1 cm^2. (b) Optical microscope image of the K-doped perovskite film.

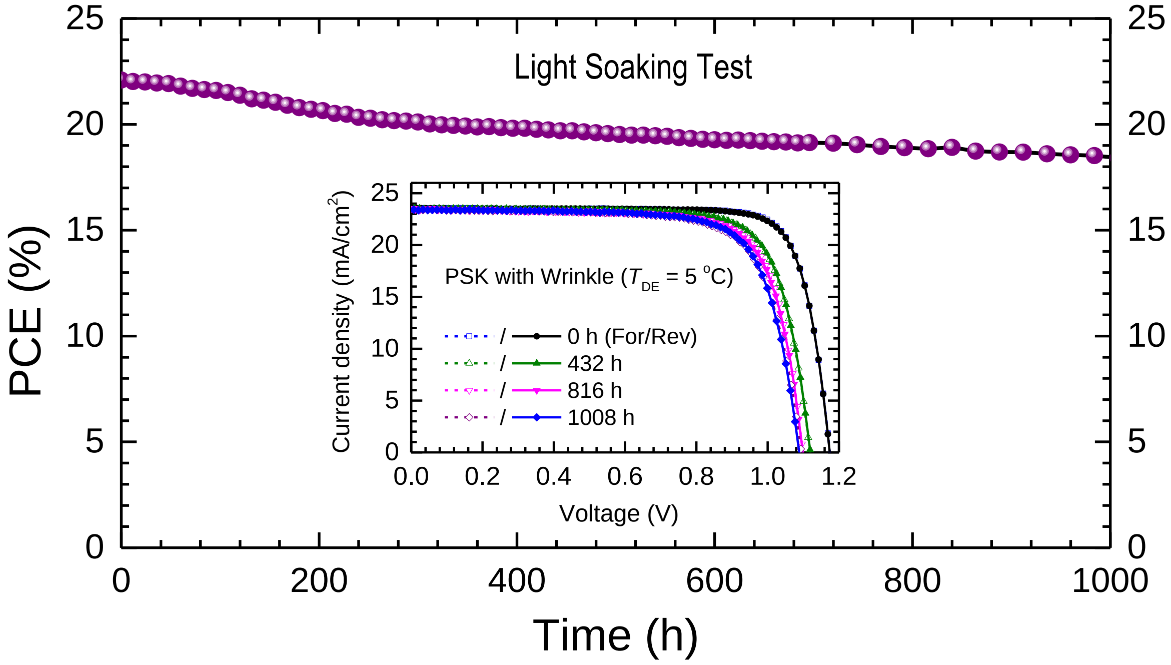

Supplementary Fig. 18. Light soaking test under continuous illumination with an intensity of 97 mW/cm^2 at temperature ranging 25-31 °C. Perovskite composition of $FA_{0.92}Cs_{0.08}PbBr_{0.15}I_{2.85}$ with KI additive formed from T_{DE} = 5 °C was employed for long-term stability measurement. The aperture area was 0.10 cm^2. Inset shows *J-V* curves measured at different light soaking time.

Supplementary Table 1. Photovoltaic parameters of short-circuit current density (J_{sc}), open-circuit voltage (V_{oc}), fill factor (FF) and power conversion efficiency (PCE) for the PSCs employing $(FAPbI_3)_{0.875}(CsPbBr_3)_{0.125}$ perovskite films formed by different T_{DE}s. T_{Sub} was 15 °C.

T_{DE} (ºC)	Scan direction	J_{sc} (mA/cm^2)	V_{oc} (V)	FF	PCE (%)
5	Forward	22.663	1.157	0.7997	20.97
	Reverse	22.661	1.159	0.8010	21.04
	Average	22.662	1.158	0.8004	21.00
15	Forward	22.484	1.139	0.7811	20.00
	Reverse	22.447	1.142	0.7848	20.12
	Average	22.466	1.141	0.7830	20.07
30	Forward	22.353	1.121	0.7721	19.35
	Reverse	22.377	1.124	0.7736	19.46
	Average	22.365	1.123	0.7729	19.41

Supplementary Table 2. Fitted parameters of fluorescence decay curves for the top surface (Top) and the bottom area (Bottom) of $(FAPbI_3)_{0.875}(CsPbBr_3)_{0.125}$ films depending on T_{DE}s.

T_{DE}	Spot*	τ (ns)	k_1 (× 10^6 s^{-1})
5 °C (Top)	T-a	136.5	3.6
	T-b	87.4	5.7
15 °C (Top)	T-c	112.2	4.4
	T-d	67.5	7.4
30 °C (Top)	T-e	72.4	6.9
5 °C (Bottom)	B-f	191.7	2.6
	B-g	162.3	3.1
15 °C (Bottom)	B-h	166.4	3.0
	B-i	159.3	3.1
30 °C (Bottom)	B-j	136.1	3.7

*Spots are marked in Fig. 4a-f in the main text.

Supplementary Note 1

Theoretical Analysis of Wrinkling of a Bilayer System

1. Calculation of the governing wavelength of the wrinkle

To describe the wrinkling of a perovskite thin film observed in experiments, we can employ a simple elastic-viscoelastic bilayer model as suggested by Im and Huang [1]. In the model, the system is modeled as an elastic layer (hereafter denoted as a subscript f)-capped viscoelastic substrate (hereafter denoted as a subscript S). Using a linear perturbation analysis, the wrinkling morphology can be approximated as a sinusoidal function with amplitude of A and period (wavelength) of λ. The development of the wrinkled morphology follows a dynamics governed by the fastest growing mode, which can be expressed with a growing constant α such that

$$\alpha \propto -\left[k^2 h_{\mathrm{f}}{}^2 - \frac{12(1-\nu_{\mathrm{f}}{}^2)\sigma_0}{E_{\mathrm{f}}}\right], k = \frac{2\pi}{\lambda} \quad (S1)$$

where h_{f} is the thickness, ν_{f} is Poisson's ratio, and E_{f} is elastic modulus of the elastic capping layer, respectively. The wrinkling results from the relaxation of the in-plane compressive stress denoted as σ_0. The origin of the in-plane stress comes from the difference of mechanical responses of the elastic capping film and the underlying viscoelastic substrate. For example, we can suggest that the difference is due mainly to the thermal expansion coefficient of the two layers [2]. The absolute value of the compressive stress developed by discrepancy of the thermal expansion coefficients can be expressed as follows.

$$|\sigma_0| = \frac{(\Delta\alpha\Delta T)E_{\mathrm{S}}}{\frac{h_{\mathrm{f}}}{H}(1-\nu_{\mathrm{S}}) + \frac{E_{\mathrm{S}}}{E_{\mathrm{f}}}(1-\nu_{\mathrm{f}})} \quad (S2)$$

where $\Delta\alpha$ denotes the difference of thermal expansion coefficients of the two layers, ν_{S}, E_{S} and H are Poisson's ratio, elastic modulus, and the thickness of the underlying viscoelastic substrate, respectively. The strain developed by the thermal expansion discrepancy is assuredly proportional to the temperature change ΔT. For most of the elastic-viscoelastic bilayer system, $\frac{E_{\mathrm{S}}}{E_{\mathrm{f}}} \ll 1$, and therefore, we can simplify eq (S2) as follows.

$$|\sigma_0| \approx \frac{(\Delta\alpha\Delta T)E_S}{(1-\nu_S)}\frac{H}{h_f}. \qquad (S3)$$

With the information on σ_0 in eq (S3), we can proceed to calculation of the fastest growing mode for the wrinkling amplitude from a relationship of $\left.\frac{\partial\alpha}{\partial k}\right|_{k=k_C} = 0, k_C = \frac{2\pi}{\lambda_C}$, where λ_C denotes the characteristic wavelength of the wrinkle corresponding to the fastest growing amplitude, which results in

$$\lambda_C = \pi\beta h_f, \beta \equiv \left(\frac{-2E_f}{3(1-\nu_f{}^2)\sigma_0}\right)^{1/2} \qquad (S4)$$

The critical compressive stress corresponding to can be calculated as follows [1],

$$\sigma_C = \left(\frac{-2h_f E_f \mu_R}{3(1-2\nu_S)(1+\nu_S)H}\right)^{1/2} \qquad (S5)$$

where μ_R denotes the rubbery modulus of the underlying viscoelastic layer. In the case in which $|\sigma_0|$ is greater than $|\sigma_C|$, the bilayer system suffers morphological instability which is initiated by small fluctuation of thickness. The small fluctuation is spontaneously evolved into the wrinkle patterns with growing amplitude. From eq (S2) and (S4), we can obtain the dependence of on the thickness of the bilayer as follows.

$$\lambda_C \propto h_f\left(\frac{h_f}{H}\right)^{1/2} \qquad (S6)$$

At equilibrium, typical wrinkle morphology exhibits amplitude which is considerably smaller than period (i.e., $A/\lambda \ll 1$). For example, in our experiments, $A/\lambda \sim 10^{-2}$. Considering this fact, we can further calculate in-plane strain of the wrinkled bilayer, ε, by calculating expanded areas of the wrinkled surface relative to the flat surface such that

$$\varepsilon = \frac{\Delta l}{\lambda} = \frac{\int_0^\lambda (1 + A^2 \sin^2 k\,x)^{1/2} dx - \lambda}{\lambda} \approx \frac{(4A+\lambda)-\lambda}{\lambda} = \frac{4A}{\lambda} \qquad (S7)$$

From eq (S3) and (S7), we can deduce an additional relationship between and thickness of the bilayer such that

$$\frac{A}{\lambda} \propto \frac{H}{h_f} \qquad (S8)$$

Using eq (S6) and (S8), it is also possible to obtain a relationship of as a function of the bilayer thickness as follows.

$$A \propto H\left(\frac{h_{\mathrm{f}}}{H}\right)^{1/2} \qquad (S9)$$

2. Effects of antisolvent temperature on the wrinkle morphology

As shown in Fig. 1d, the major factor governing the wrinkle morphology of a perovskite thin film is the temperature of antisolvent diethyl ether (T_{DE}). At fixed temperature of the substrate (T_{Sub}), we observed that λ increases while A decreases with increasing T_{DE}. Using the bilayer model suggested in the previous part, we can explain these dependences on T_{DE}. The role of the antisolvent is to drive solvent (i.e., DMSO) out of the spin-coated perovskite precursor film by inducing phase separation. For the sake of simplicity, let us assume that the spin-coated layer form bilayer mixture such that DMSO solution containing perovskite precursors and DE. Due to the limited miscibility of DMSO and DE, the mixture suffers phase separation, and the separation can be modeled as spinodal decomposition. For a simple binary mixture which suffers thermodynamic instability, the free energy density of the mixture, ΔF, can be modeled as a function of the composition of one of the components, ϕ [3],

$$\Delta F = \gamma\phi(1-\phi) + k_{\mathrm{B}}T(\phi\log\phi + (1-\phi)\log(1-\phi))$$

where γ denotes a constant concerning the interaction energy of two components and k_{B} is the Boltzmann constant. In a typical temperature (T)-composition (ϕ) phase diagram of a binary mixture, spinodal decomposition results in two phases containing high and low compositions. In the case of DMSO solution and DE binary mixture, the molar fraction of DMSO solution in the DMSO-rich phase and the DE-rich phase can be expressed as $1-\phi_{\mathrm{d}}$ and ϕ_{d}, respectively. Considering a fact that DMSO-solution has higher density, the separated phases form bilayer composed of upper layer containing dilute DMSO-solution and lower layer containing higher concentration of DMSO-solution. Without losing generality, for DMSO-dilute phase, the (upper) elastic film thickness would be proportional to ϕ_{d}, while the (lower) viscoelastic layer thickness would be proportional to $1-\phi_{\mathrm{d}}$. Therefore, using eq (S6) and (S9), we can proceed to expression of λ and A as a function of ϕ_{d} as follows

$$\lambda_C \propto h_f\left(\frac{h_f}{1-\phi_d}\right)^{1/2} \propto (1-\phi_d)^{-1/2}, A \propto (1-\phi_d)\left(\frac{h_f}{1-\phi_d}\right)^{1/2} \propto (1-\phi_d)^{1/2} \qquad (S10)$$

In eq (S10), we assumed that the thickness of the initially formed elastic layer is not dependent of ϕ_d.

Based on a typical phase diagram of spinodal decomposition, we can find that ϕ_d increases as the temperature of the binary mixture increases approaching the critical temperature. Therefore, ϕ_d for the case in which DE temperature is relatively low (i.e., T_{DE} = 5 °C) is smaller than ϕ_d for the cases of relatively high DE temperature (i.e., T_{DE} = 15 °C). Then, from eq (S10), we can find that the lower the value of T_{DE}, the lower the value of ϕ_d, and therefore, λ_C increases while A decreases. This can explain the experimental observation of the changes of λ_C and A with different T_{DE}, as reported in Fig. 1d.

3. Effect of the composition of perovskite materials on the wrinkling

We observed that the substitution of FA with Cs or MA and I with Br resulted in the decrease in λ and the increase in A at a certain substitution ratio. The smaller size of the substituents can increase σ_0, which increase λ according to eq (S3) and (S4). Regarding the increased A, η is decreased with increasing the amount of Cs and Br or MA and Br (see Fig. 1b and c). According to ref S12, amplitude (A) is derived function of dimensionless growth rate (s), characteristic time scale (τ) and formation time (t) ($A = A_0 e^{\frac{st}{\tau}}$, s = α-μ_R/E_f and τ = η/E_f). The A is exponentially anti-proportional to η. Therefore, when η is decreased, A is enlarged. Except for the specific ratio, however, the compositions with $z \geq 0.25$ or $x \geq 0.8$ formed a solid bottom layer, which leads to a very large η (see Supplementary Fig. 6) and thereby a significant increase of characteristic time scale (τ) to about 10^4~10^5 times, resulting in less formation of wrinkled texture.

4. Effect of the annealing condition on the wavelength of the wrinkles

Given a condition of $\sigma_0 > \sigma_c$, wrinkling starts with long wavelength (λ_0) which will be eventually narrowed and saturated as the stress is being relaxed until $\sigma_0 = \sigma_c$ [4]. However, in perovskite film formation process, λ_0 cannot be saturated because the bottom layer is solidified before it is saturated, which may lead to a residual compressed stress after spin-coating [5]. The slight decrement of λ after annealing is evidence of the presence of residual stress because the

relaxation of residual stress will further decrease λ as shown in Supplementary Fig. 2d-e.

5. Null contribution of E_f and v_f

E_f and v_f can be also assumed to be constant due to a small difference in E_f between 10.2~11.8 GPa for $FAPbI_3$ and 9.7~12.3 GPa for $FAPbBr_3$ even upon replacing iodide with bromide [6] and small v_f of perovskite (0.28~0.33) [7].

6. Effect of T_{Sub}

At fixed temperature such as T_{DE} = 15 °C, λ increases, while A decreases with increasing T_{Sub} from 5 °C to 15 °C (see Fig. 1d). Upon increasing T_{Sub}, h_f is expected to increase because the miscibility between DMSO and diethyl ether is enhanced by elevating T_{Sub}. This can lead to an increase in h_f but decreases in A.

Supplementary Note 2

The PL peak position E_0 and width σ were extracted by fitting each frame of the PL data with a hyperbolic secant, according to $\mathrm{PL}(E) = I_0\left[\exp\left(-\frac{E-E_0}{\sigma}\right) + \exp\left(\frac{E-E_0}{\sigma}\right)\right]^{-1}$. The band edge energy was determined by fitting the band edge of the spectrum at the end of each measurement, to all prior spectra, where the fitted spectrum was allowed to be shifted in energy and scaled in intensity. For comparison with PL, the band edge was shifted in energy to match the final PL peak position.

The PL peak position as well as the band edge energy continuously shift to lower energies upon increasing perovskite layer thickness. Above approx. 100 nm (longer times), the shift of the PL peak position and band edge are nearly identical, and the PL peak width is constant. Below approx. 100 nm (shorter times), the shift of the PL peak is steeper and accompanied by a pronounced decrease of the PL peak width. A shift of the band gap / PL peak to higher energies, together with an increased PL peak width are characteristic for a quantum confinement effect, which decreases with increasing layer thickness [8-12]. We note that the thickness of 100 nm, which is where the decrease of σ stops, is far above the confinement limit reported for halide perovskites (~ 25-30 nm) [13-15]. This suggests that even though the average layer thickness is above the confinement limit, individual grains can still be very small and can have limited electronic interaction with adjacent grains. The PL quantum efficiency of such grains is

enhanced by the quantum confinement effect [16], so that they contribute overproportionally strong in the PL spectra. The slower shift for longer time could either stem from a change in stoichiometry or a decreasing confinement effect. Since the PL peak width remains constant during this shift, we can exclude a decreasing confinement effect as cause for this spectral shift, so we associate it with a change in stoichiometry during spin coating (not annealed film). This indicates a preferential formation of a bromine-rich phase, possibly e.g. due to differences in the enthalpy of formation, as reported for $MAPbI_3$ and $MAPbBr_3$ [17], or different diffusivity in solution of the different compounds. By comparison with another mixed halide perovskite, $MAPbI_{3-y}Br_y$ [18], we are able to estimate the change in stoichiometry during spin coating. The observed shift of the band edge and PL peak position by about 50 meV corresponds to a change from $y = 0.5$ to $y = 0.375$, which would correspond to a change of material composition from $(FAPbI_3)_{0.833}(CsPbBr_3)_{0.167}$ to $(FAPbI_3)_{0.875}(CsPbBr_3)_{0.125}$ in our case, (based on the assumption that the latter is the final film stoichiometry).

Supplementary Note 3

Evolution of the wrinkle pattern of the bilayer

To confirm the wrinkling mechanism of a bilayer structure observed in our experiments, we provide a computer simulation of the temporal morphological evolution of the surface wrinkles of the bilayer. For this simulation, we employed a typical finite-difference method for 2D simulation box (800×800) with periodic boundary condition. According to the theoretical and numerical scheme suggested by Im and Huang [1], we modeled the morphological evolution of the bilayer wrinkles as shown in Supplementary Fig. 7. As shown in Supplementary Fig. 8, one can find that the simulated wrinkle morphology is similar to the experimentally observed morphology. The similarity is confirmed again by comparing the 2D fast Fourier transform (2D FFT) signals obtained from the simulated and experimentally observed morphologies, in which isotropic wrinkles pattern with notable concentric ring patterns which correspond to the characteristic length scale (i.e., λ_C) of the wrinkles. The computer simulated bilayer wrinkle morphology strongly supports that the wrinkling mechanism of the perovskite thin film hinges on the relaxation of the in-plain compressive stress developed in the elastic-viscoelastic bilayer.

Supplementary Note 4

Analysis of charge carrier density from PL intensity

The charge carrier recombination in halide perovskites is given by

$$\frac{dn(t)}{dt} = -\, k_1 n(t) - k_2 n(t)^2 - k_3 n(t)^3, \qquad \text{(S11)}$$

where k_1 is associated with mono-molecular, trap-assisted recombination, k_2 is the rate constant for bimolecular and in this case radiative recombination, and k_3 is the rate constant for Auger-recombination. In general, this leads to a non-exponential decrease of the charge carrier density and accordingly of the PL intensity, which is proportional to

$$\mathrm{PL}(t) \propto k_2 n(t)^2. \qquad \text{(S12)}$$

However, for low charge densities, i.e. for low excitation densities or long delay times, the mono-molecular recombination becomes dominant and the change of charge carrier density is given by

$$\frac{dn(t)}{dt} \approx -k_1 n(t). \qquad \text{(S13)}$$

Thus, the temporal evolution of the charge carrier density is given by

$$n(t) \approx n_0 \exp(-k_1 t). \qquad \text{(S14)}$$

Inserting this into eq. S12 yields a mono-exponential decay of the PL intensity according to

$$\mathrm{PL}(t) \propto k_2 n(t)^2 \approx k_2 n_0^2 \exp(-2k_1 t). \qquad \text{(S15)}$$

The PL decay for low charge carrier densities thus depends on the mono-molecular recombination constant and contains information about the trap density in the material.

Supplementary References

1. S. H. Im, R. Huang, Evolution of Wrinkles in Elastic-Viscoelastic Bilayer Thin Films. *J. Appl. Mech.* **2005**, 72, 955−961.
2. W. -J. Chang, T. -H. Fang, C. -M. Lin, Thermally induced viscoelastic stresses in multilayer thin films, *J. Appl. Phys.* **2005,** 97, 103521.

3. Hill, T. L. An Introduction to Statistical Thermodynamics; Dover Publications, Inc.: New York, 1986.

4. S. Chatterjee, C. McDonald, J. Niu, S. S. Velankar, P. Wang, R. Huang, Wrinkling and folding of thin films by viscous stress. *Soft Matter*, **2015**, 11, 1814–1827.

5. K. A. Bush, N. Rolston, A. Gold-Parker, S. Manzoor, J. Hausele, Z. J. Yu, J. A. Raiford, R. Cheacharoen, Z. C. Holman, M. F. Toney, R. H. Dauskardt, M. D. McGehee. Controlling Thin-Film Stress and Wrinkling during Perovskite Film Formation. *ACS Energy Lett*. **2018**, 3, 1225−1232

6. S. Sun, F. H. Isikgor, Z. Deng, F. Wei, G. Kieslich, P. D. Bristowe, J. Ouyang, A. K. Cheetham, Factors Influencing the Mechanical Properties of Formamidinium Lead Halides and Related Hybrid Perovskites. *ChemSusChem*, **2017**, 10, 3740−3745.

7. J. Feng, Mechanical properties of hybrid organic inorganic $CH_3NH_3BX_3$ (B = Sn, Pb; X = Br, I) perovskites for solar cell absorbers. *APL Mater*. **2014**, 2, 081801.

8. V. Malgras, S. Tominaka, J. W. Ryan, J. Henzie, T. Takei, K. Ohara, Y. Yamauchi, Observation of Quantum Confinement in Monodisperse Methylammonium Lead Halide Perovskite Nanocrystals Embedded in Mesoporous Silica. *J. Am. Chem. Soc.* **2016***,* 138, 13874−13881.

9. M. Anaya, A. Rubino, T. C. Rojas, J. F. Galisteo-López, M. E. Calvo, H. Míguez, Strong Quantum Confinement and Fast Photoemission Activation in CH3NH3PbI3 Perovskite Nanocrystals Grown within Periodically Mesostructured Films. Adv. *Optical Mater.* **2017**, 5, 1601087.

10. M. E. Kamminga, H.-H. Fang, M. R. Filip, F. Giustino, J. Baas, G. R. Blake, M. A. Loi, T. T. M. Palstra, Confinement Effects in Low-Dimensional Lead Iodide Perovskite Hybrids.

Chem. Mater. **2016**, 28, 4554−4562.

11. F. Zhang, S. Huang, P. Wang, X. Chen, S. Zhao, Y. Dong, H. Zhong, Colloidal Synthesis of Air-Stable CH3NH3PbI3 Quantum Dots by Gaining Chemical Insight into the Solvent Effects. *Chem. Mater*. **2017**, 29, 3793−3799.

12. M. Chauhan, Y. Zhong, K. Schötz, B. Tripathi, A. Köhler, S. Huettner, F. Panzer, Investigating two-step MAPbI3 thin film formation during spin coating by simultaneous in situ absorption and photoluminescence spectroscopy. *J. Mater. Chem. A* **2020**, 8, 5086–5094.

13. D. N. Dirin, L. Protesescu, D. Trummer, I. V. Kochetygov, S. Yakunin, F. Krumeich, N. P. Stadie, M. V. Kovalenko, Harnessing Defect-Tolerance at the Nanoscale: Highly Luminescent Lead Halide Perovskite Nanocrystals in Mesoporous Silica Matrixes. *Nano Lett.* **2016**, 16, 5866−5874.

14. E. S. Parrott, J. B. Patel, A.-A. Haghighirad, H. J. Snaith, M. B. Johnston, L. M. Herz, Growth modes and quantum confinement in ultrathin vapour-deposited $MAPbI_3$ films. *Nanoscale* **2019**, 11, 14276–14284.

15. L. Polavarapu, B. Nickel, J. Feldmann, A. S. Urban, Advances in Quantum-Confined Perovskite Nanocrystals for Optoelectronics. *Adv. Energy Mater.* **2017**, 7, 1700267.

16. S. Kan, T. Mokari, E. Rothenberg, U. Banin, Synthesis and size-dependent properties of zinc-blende semiconductor quantum rods. *Nat. Mater.* **2003**, 2, 155–158.

17. C. Rehermann, A. Merdasa, K. Suchan, V. Schröder, F. Mathies, E. L. Unger, Origin of Ionic Inhomogeneity in MAPb(IxBr1−x)3 Perovskite Thin Films Revealed by In-Situ Spectroscopy during Spin Coating and Annealing, *ACS Appl. Mater. Interfaces* **2020**, 12, 30343−30352.

18. C. M. Sutter-Fella, Y. Li, M. Amani, J. W. Ager, F. M. Toma, E. Yablonovitch, I. D. Sharp, A. Javey, High Photoluminescence Quantum Yield in Band Gap Tunable Bromide Containing Mixed Halide Perovskites. *Nano Lett*. **2016**, 16, 800−806.
