# Supplementary material for: How antisolvent miscibility affects perovskite film wrinkling and photovoltaic properties
Source: Nat Commun. 2021 Mar 10;12:1554. doi: 10.1038/s41467-021-21803-2 (PMC7946869; doi:10.1038/s41467-021-21803-2)
Supplement: Supplementary file 3 — Solar Cells Reporting Summary [file 41467_2021_21803_MOESM3_ESM.pdf]

nature research

Corresponding author(s): Nam-Gyu Park

Solar Cells Reporting Summary

Nature Research wishes to improve the reproducibility of the work that we publish. This form is intended for publication with all accepted papers reporting the characterization of photovoltaic devices and provides structure for consistency and transparency in reporting. Some list items might not apply to an individual manuscript, but all fields must be completed for clarity.

For further information on Nature Research policies, including our data availability policy, see Authors & Referees.

Experimental design

Please check: are the following details reported in the manuscript?

1. Dimensions

Item	Yes	No	Details
Area of the tested solar cells	☒	☐	0.125 cm2 or 0.1 cm2 (in Experimental section)
Method used to determine the device area	☒	☐	Defined by the aperture

2. Current-voltage characterization

Item	Yes	No	Details
Current density-voltage (J-V) plots in both forward and backward direction	☒	☐	Fig 3a and Fig s17a
Voltage scan conditions<br>*For instance: scan direction, speed, dwell times*	☒	☐	J-V curves were measured at a scan rate of 130 mV/s or 260mV/s
Test environment<br>*For instance: characterization temperature, in air or in glove box*	☒	☐	All device are measured in air at room temperature
Protocol for preconditioning of the device before its characterization	☐	☒	No preconditioning is required before characterization.
Stability of the J-V characteristic<br>*Verified with time evolution of the maximum power point or with the photocurrent at maximum power point; see ref. 7 for details.*	☒	☐	Fig s17a

3. Hysteresis or any other unusual behaviour

Item	Yes	No	Details
Description of the unusual behaviour observed during the characterization	☐	☒	No Unusual behavior (Fig 3a and Fig s17a)
Related experimental data	☐	☒	No Unusual behavior

4. Efficiency

Item	Yes	No	Details
External quantum efficiency (EQE) or incident photons to current efficiency (IPCE)	☒	☐	Fig 3b
A comparison between the integrated response under the standard reference spectrum and the response measure under the simulator	☒	☐	Fig 3b
For tandem solar cells, the bias illumination and bias voltage used for each subcell	☐	☒	Single-layer solar cell

5. Calibration

Item	Yes	No	Details
Light source and reference cell or sensor used for the characterization	☒	☐	Current density-voltage (J-V) curves were measured under AM 1.5G one sun (100 mW/cm2) illumination using a solar simulator (Oriel Sol 3A, class AAA) equipped with 450 W Xenon lamp (Newport 6280NS) and a Kiethley 2400 source meter.
Confirmation that the reference cell was calibrated and certified	☒	☐	The light intensity was adjusted by NREL-calibrated Si solar cell having KG-5 filter.

Calculation of spectral mismatch between the reference cell and the devices under test	☒ Yes ☐ No	It was reflected to the calibration process.

6. Mask/aperture

Size of the mask/aperture used during testing	☒ Yes ☐ No	0.125 cm2 or 0.1 cm2 (in Experimental section)
Variation of the measured short-circuit current density with the mask/aperture area	☐ Yes ☒ No	0.125 cm2 for most of devices , 0.1cm2 for only best performing devices

7. Performance certification

Identity of the independent certification laboratory that confirmed the photovoltaic performance	☐ Yes ☒ No	It has not measured in the independent certification laboratory
A copy of any certificate(s)<br>*Provide in Supplementary Information*	☐ Yes ☒ No	We don't have a certification.

8. Statistics

Number of solar cells tested	☒ Yes ☐ No	18 cells (in Fig s10)
Statistical analysis of the device performance	☒ Yes ☐ No	(in Fig s10)

9. Long-term stability analysis

Type of analysis, bias conditions and environmental conditions<br>*For instance: illumination type, temperature, atmosphere humidity, encapsulation method, preconditioning temperature*	☒ Yes ☐ No	Fig s17a
